# Establishing content validity in a novel patient reported outcome measure for cardiac arrhythmia ablation patients

**DOI:** 10.1186/s12955-015-0233-5

**Published:** 2015-03-20

**Authors:** Kathleen L Withers, Kathryn A Wood, Grace Carolan-Rees, Hannah Patrick, Mauro Lencioni, Michael Griffith

**Affiliations:** Cedar, Cardiff & Vale University Health Board, Cardiff Medicentre, Heath Park, Cardiff, CF14 4UJ UK; Duke University School of Nursing, 307 Trent Drive, DUMC 3322, Durham, NC 27710 USA; National Institute for Health and Care Excellence, 10 Spring Gardens, London, SW1A 2BU UK; University Hospitals Birmingham NHS Foundation Trust, New Queen Elizabeth Hospital, Birmingham, B15 2GW UK

**Keywords:** Cardiac arrhythmias, PROMs, Patient Reported Outcome Measures, Qualitative Research, Questionnaire Validation

## Abstract

**Aim:**

Preliminary content validity testing of a UK Patient Reported Outcome Measure (PROM) for use in cardiac arrhythmia patients undergoing ablation treatment.

**Methods:**

Twenty five patients diagnosed with symptomatic cardiac arrhythmias participated in qualitative interviews to obtain their perspective of a draft PROM. As part of the process to establish preliminary content validity, patients were asked to complete the questionnaires and to identify missing and redundant items within the PROM, while also reviewing the instructions and formatting. The questionnaires were updated iteratively to reflect patient feedback.

**Results:**

Recurring themes were identified during qualitative interviews leading to improvements to the tool. Following modification of the PROM, based on patient feedback, subjects reported that the tool was fully inclusive and easy to comprehend. Patients found the instructions and layout of the tool acceptable and easy to use.

**Conclusion:**

Qualitative patient interviews are an important part of PROM tool development. In the case of this cardiac ablation PROM, it enabled end users to assess the tool for inclusivity and accessibility, and to ensure that it addressed concerns important to the patient. Cognitive interviews were able to obtain patients’ perspectives to establish face validity and content validity of the PROM. This is part of a process which will ensure that this disease-specific PROM measures cardiac arrhythmia patient symptoms and impact on patients’ lives accurately and sensitively. The next study will use the PROM prospectively in over 450 arrhythmia patients to prospectively validate the tool.

**Condensed abstract:**

Patients diagnosed with symptomatic cardiac arrhythmias provided feedback through cognitive interviews to facilitate improvements in a new disease specific PROM establishing preliminary face and content validity.

## Introduction

The 2008 report “High Quality Health Care for All” by the Health Minister Lord Darzi [1] highlighted the importance of assessing the effectiveness of health care from the patient perspective, and suggested that this should be measured through routine collection of Patient Reported Outcome Measures (PROMs). A national programme to collect PROM data from patients receiving hip or knee replacements, varicose vein surgery or groin hernia surgery was established in April 2009. A subsequent UK Government White Paper published in 2010 confirmed the importance of PROMs within the National Health Service (NHS), and announced plans to extend their use wherever practical to other procedural interventions [2].

Gathering outcome feedback from patients in the form of PROMs has several purposes, not least in assessing whether the intervention provided actually improved the patients health or wellbeing. While a healthcare provider may judge a procedure or treatment to be a success using objective clinical measures, efforts should be made to assess whether this reflects an improvement in the patients’ perspective of their symptoms and quality of life. The aim is to use PROMs to support commissioning within the NHS, based on robust, patient centred evidence to fund those services with proven patient benefit. Patient responses should allow providers to benchmark their performance outcomes.

As the use of PROMs becomes more widespread within certain diseases, research is underway to identify other clinical areas where PROMs collection may be feasible. One area where researchers are currently aiming to develop the use of PROMs is in symptomatic cardiac arrhythmia patients who undergo ablation treatment. Cardiac arrhythmias occur sporadically and cause a range of symptoms including palpitations, shortness of breath, fatigue and dizziness, which can have a significant detrimental effect on the quality of life of sufferers. Cardiac ablation is a widely-used treatment option for symptomatic arrhythmias which are unresponsive to drug therapy, and several guidelines have been published on its use [3-13]. Cardiac ablation aims not only to improve outcome, but also to reduce or stop symptoms [9]; this can be most accurately assessed by the patients themselves, making PROMs an ideal tool for evaluating the clinical benefit of the procedure. PROMs tools are already available for some types of arrhythmias including atrial fibrillation [14] and supraventricular tachycardia [15]. To date, no tool has been validated in a UK population which combines measures of treatment expectation and experiences with symptom impact and severity in patients with any symptomatic arrhythmia. Such a tool allows data to be gathered to facilitate comparative analysis, improve knowledge and inform clinical practice.

This paper describes the methods used to establish preliminary validity of our PROM for a population treated with ablation of cardiac arrhythmias. Using responses gained via face to face patient interviews we aimed to improve the PROM iteratively by ensuring that the tool is fully inclusive of aspects of the disease important to patients and easy to comprehend and complete. These are important steps which will confirm face validity, ensuring it is clear and easy to read and understand. This allowed us to establish preliminary content validity of the tool to ensure it measures all aspects of the disease and treatment most important from a patient perspective.

### Study aim

As part of an iterative process to further develop and validate several questionnaires used in a pilot study to form a single PROM tool, a multicentre, prospective observational study was carried out after approval by the Nottingham 1 Research Ethics Proportionate Review Sub-Committee. This paper reports on the first stages of the study which involved face- to- face interviews with arrhythmia patients, to begin the process of establishing content validity of the PROM tool. Our objective was to identify areas of the tool which required additional clarification; adding items deemed to be relevant to the patient population, and removing redundant items. The instructions and layout of the items on each questionnaire were also investigated to clarify wording and improve usability.

### Retrospective pilot – methods and results

As has been previously published [16], a retrospective study was carried out at three clinical sites (University Hospital Wales, Cardiff; Queen Elizabeth Hospital, Birmingham; and Freeman Hospital, Newcastle) to determine the feasibility of administering PROMs in patients undergoing ablation treatment for a variety of symptomatic cardiac arrhythmias. The original tool comprised of a battery of three questionnaires: i) a new short, arrhythmia specific, patient expectation questionnaire developed by our multidisciplinary team; ii) an adaptation of the arrhythmia disease specific “Patient Perception of Arrhythmia” questionnaire (PPAQ) developed by Wood et al. [15]; iii) the EuroQol EQ-5D-5L questionnaire [17,18], and was sent out to 791 patients. A response rate of over 74% (n = 584) was achieved including 569 analysable responses, with a high proportion of participants reporting an improvement in symptoms and quality of life following ablation [16].

### Prospective study – phase 1: PROM development and qualitative interviews

Data from the retrospective pilot study allowed a draft cardiac arrhythmia PROM tool to be developed. Questionnaire responses, feedback from free text observations and comments during telephone enquiries provided useful data to the study team for revisions to make the PROM more effective for future studies. Replies indicated that while most areas of the questionnaires appeared to be well understood, some instructions and items required clarification. Additionally, comments suggested that further items should be considered to ensure all relevant areas which are important to patients were included.

Changes were made to the questionnaires based on feedback received to produce a PROM with improved readability and clarity. Additionally, some new items were included to gather more information on fields such as co-morbidities, arrhythmia medication and complications following the procedure.

### Patient enrolment

Patients under the care of physicians at two clinical sites (University Hospital Wales, Cardiff and Queen Elizabeth Hospital, Birmingham) were eligible for inclusion in this stage of the study if they had a diagnosis of symptomatic cardiac arrhythmia and were awaiting for, and/or had received a cardiac ablation. Patients were required to be at least 18 years of age, able to communicate in English or Welsh and willing to participate in interviews lasting approximately 1.5-2 hours.

Patients were initially approached by their treating physician and given a patient information sheet which explained the study. Those who were interested in taking part in interviews were asked to complete a permission sheet which authorised a researcher to contact them to discuss their involvement. Interested patients were given further details of the study and an appointment for the interview. In one case where the researcher was on-site, a patient wished to carry out the interview immediately following their clinic appointment. In all other cases the patients were given the opportunity to choose the time and the place of their interview. In 21 cases these interviews took place in the patients’ own homes; four, including the case above took place in a quiet room at a convenient hospital site.

Purposive sampling was used to obtain a representative selection of participants that included both sexes, a range of arrhythmia substrates and a wide age range of patients. Consecutive patients who met the sampling criteria were approached at four separate clinics. A total of 41 patients were invited to participate in the study, eleven declined or did not respond following initial contact. Of the remainder, two were subsequently withdrawn as their ablation procedure was cancelled and three were not required as data saturation had been reached. The remaining 25 patients took part in the interview process, 11 of these were receiving treatment in Cardiff, while 14 were under the care of physicians in Birmingham.

Prior to the interviews, study procedures were explained and informed written consent was obtained. All interviews were carried out by a single trained researcher (KLW) using a semi-structured interview guide, and were audio-taped with the patients’ permission (one patient declined, two were not recorded due to technical issues). The interview guide used a list of questions (developed by Withers et al. [16]) to direct the process. “Think aloud” techniques were used to elicit patient opinions on the PROM, with patients invited to read each question aloud, explain their understanding of the question and describe any areas which lacked clarity. Areas identified by the patient as being unclear were probed in detail and patients were asked to suggest improvements. At the end of the session, participants were asked if there were any topics they had expected to see that weren’t covered, or issues which needed to be discussed in more detail. They were also encouraged to discuss problems they experienced as a result of their arrhythmia. Recorded sessions lasted between 23 and 93 minutes (mean duration of 50 minutes). The interviews were transcribed verbatim, checked for accuracy and accompanied by field notes and annotated questionnaires. Additional interviews were carried out until the research team found no new issues were being identified, suggesting data saturation had been reached.

**Figure 1:**
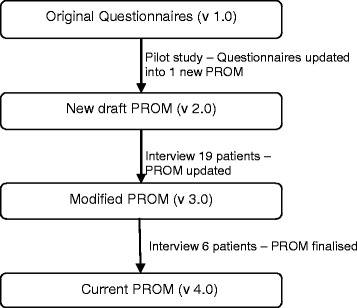
**Steps taken during PROM development.**

The process was iterative and used grounded theory methodology throughout the interviewing process, with information from initial interviews used to inform and guide later ones. The research team monitored responses and used content analysis to identify common themes from the qualitative data. Following interviews with 19 patients, the PROM was revised to reflect the feedback received. These changes were then tested in a further six patients. Figure 1 illustrates the steps taken in developing the PROM.

## Results

### Participant characteristics

The cohort of 25 individuals was selected from a population of patients who were awaiting, or had received an ablation for a symptomatic cardiac arrhythmia. The majority (n = 15; 60%) were female with an age range of 43–87 years (average 61.8 years); where known, the average length of time since diagnosis was 3.24 years (range 11 months – 19 years). At the time of interview, 14 patients (56%) had received an ablation procedure, and four of these (16%) were awaiting a repeat ablation. Eleven participants (44%) were awaiting their first ablation procedure. Patients had one of four primary diagnoses as detailed in Table 1.

**Table 1:** **Number of patients across arrhythmia substrates interviewed before and after their ablation procedure**

**Interviewed:**	Total*	**Atrial fibrillation (AF)**	**Supraventricular tachycardia**	**Atrial flutter**	**Ventricular ectopics**
Pre ablation (male/female)	15 (5/10)	9 (4/5)	2 (0/2)	3 (1/2)	1 (0/1)
Post ablation (male/female)	14 (8/6)	10 (6/4)	2 (0/2)	2 (2/0)	0 (0/0)

*Total sample size = 25 patients (15 female / 10 male). Four patients who had previously received an ablation for paroxysmal AF were also awaiting a further ablation procedure for either paroxysmal AF (3 patients: 1 male, 2 female) or persistent atrial flutter (1 female patient). These patients were therefore classed as both pre and post-procedure at the time of interview.

### Interview results

The initial two questions relating to the “frequency” and “duration” of episodes of arrhythmia were reported by all initial interviewees as difficult to distinguish between, and the word “duration” was not well understood. Patients felt that “length” (of arrhythmia attacks) was a better option, and suggested, the words “frequency” and “length” were typed in bold capitals with a short descriptive sentence for each, i.e. “how often they occur” for frequency and “how long they last” for length [19,20]. Following these updates, no problems were encountered on these questions in subsequent interviews.

The two parts of question 3 (what do) “you expect to happen to your tiredness and breathlessness” were generally well understood, but several suggestions were made to improve the layout. These included making key words stand out by using uppercase or bold lettering. Some of the first patients interviewed also stated that while they expected symptoms such as tiredness and breathlessness to potentially worsen immediately after the procedure, they would expect them to improve in the longer term. This led to the subjects requesting that the phrase “after you have recovered from the procedure” be added to these and the previous questions to clarify the time frame under discussion. These changes during the initial stages of the study resolved issues with this field.

The following two questions (Questions 4 and 5) relating to past procedures did not cause any confusion to the participants, and were easily understood requiring no adaptation. Discussion of Question 6, a table of symptoms suggested that no items were redundant, but some new areas were identified for inclusion. This led to five additional items, while terms used in five existing fields were expanded to fully describe symptom variations (Table 2). This ensured that symptoms important to patients such as anxiety, tiredness and difficulty sleeping were included.

**Table 2:** **Amended post interview question 6 table (amendments in bold)**

	**0 None**	**1 Mild**	**2 Moderate**	**3 Severe**
Palpitations/fast or irregular heartbeats	0	1	2	3
Heart flutters	0	1	2	3
**Extra heart beats/missed heart beats**	0	1	2	3
Fatigue/no energy	0	1	2	3
Dizziness/light-headedness**/feeling faint**	0	1	2	3
Hard to catch breath**/short of breath**	0	1	2	3
Chest pressure as heart is racing	0	1	2	3
Headache**/migraine**	0	1	2	3
Trouble concentrating	0	1	2	3
Neck pounding**/neck pain/neck discomfort**	0	1	2	3
Passing out/fainting**/blackouts**	0	1	2	3
**Trouble sleeping**	0	1	2	3
**Tiredness/sleepiness**	0	1	2	3
**Nausea/vomiting**	0	1	2	3
**Anxiety/fear/worry**	0	1	2	3

Feedback related to Questions 7 and 8, discussing how often episodes of palpitations occur and how long they last, suggested that patients would naturally indicate more than one answer. This was clarified by specifying that only one could be chosen. Whilst feedback suggested this would not always resolve the issue, patients felt that it would help in most circumstances. Wording was also updated to reflect that the PROM team wanted feedback relating to the “usual” frequency and length of attacks. Several participants felt that the maximum duration of palpitations of 1 hour was inadequate, and following expert clinical input, the options were updated to reflect those patients with longer lasting episodes of arrhythmia. While some patients also felt that they would like a free-text option, or wider choice for the frequency of attacks, it was considered impractical to expand the options further without impacting on the wider PROM design, while free-text would require interpretation by a third person.

Question 9 (impact on social activities in the last 30 days) was appropriate to subjects, while Question 10 (work/school/college days affected) was problematic only for those patients in voluntary work or acting as care-giver. Wording was subsequently inserted in the instruction to assert that these roles should be included. The three questions were reordered with work being placed first, and in each of the three, a “not applicable” option was added as some interviewees felt this was a more appropriate response in some instances. Several subjects felt that the time period of 30 days was too short a time period for consideration, as they noted that arrhythmia patients often have “good” and “bad” months, and a 30 day cut off may not give a full representation. However, overall, participants felt that this was a suitable time period and was not too difficult to remember, while a longer time frame could lead to recall issues.

The draft PROM (v 2.0) asked patients to specify the number of times they had needed to visit their GP/Hospital in the 30 days prior to the procedure. Several patients felt that this should be two separate questions, with some noting that the costs of the two services are very different. The question was subsequently modified in version 3.0 to reflect this feedback.

The table of questions (Q13) relating to impact on life produced numerous comments, with many suggestions for additional items. While some of these were suggested by only one patient, several themes were recurring, and four fields were subsequently added. These were: i) impact on sport and leisure activities; ii) impact on family and friends; iii) reduced confidence, and iv) financial impact (due to time off work, childcare costs etc.). All existing fields were felt to be appropriate, with the only additional change being to alter “Disagree” to “Not Applicable” as a column header.

The new fields which had been added to the PROM to gather data on arrhythmia medication and concomitant illnesses were generally well accepted, with only slight changes required to the wording to reiterate that only arrhythmia related drugs were to be listed and to clarify that the illnesses listed could be caused by their arrhythmia, but were for background purposes only. As the pre and post-procedure PROMs are very similar, issues were generally the same for the post-procedure tool as for the pre-procedure tool, with one main difference whereby Question 2 (relating to the duration of palpitations) was updated to add a “Stopped” option, as participants felt that this was clearer than asking patients to omit the question if not applicable.

Regarding complications, several patients reported that they had not been warned of the possibility that the arrhythmia could get worse following ablation. This is not classed as a clinical complication, and as symptom severity is included elsewhere in the tool, it was not added to the PROM as a separate field in the complication table.

All participants felt that the layout of the final drafts of the pre and post procedure questionnaires [19,20] were good, and the font size clear and easy to read. The current version of the PROMs are available online at: http://www.cedar.wales.nhs.uk/ccap. Other than the changes discussed, the questions were correctly interpreted by patients with very few issues. Overall subjects commented that they felt the tool was highly comprehensive and covered all relevant areas, with clear instructions throughout.

## Discussion

Patient feedback and cognitive interviews as described in this paper added valuable data to establish preliminary content validity for a PROM to be used in the evaluation of patient outcomes following cardiac ablation. Our research identified several important concepts for inclusion in the tool including items focused on trouble sleeping and anxiety related to the arrhythmia as well as the disease's impact on family. Some of these issues have been poorly reported in previous literature and can significantly impair quality of life. Items were also added to reflect patient concerns related to the arrhythmia’s impact on their sport and leisure activities; reduced confidence, and financial implications of the arrhythmia (e.g. due to time off work). While some of the issues identified such as distress and lack of support have been previously reported in the literature [21-24], this study highlights those issues which are most problematic for this patient group, allowing them to be prioritised for inclusion in the PROM.

The qualitative interviews allowed participants to verbally explore areas which were important to them without being limited to pre-determined options and wording. The “think aloud” technique enabled the researcher to identify areas within the tool which were difficult for patients to understand or interpret. Probing questions identified the key issues and allowed participants to suggest suitable improvements. This ensured that the words and phrases used in the PROM are easy to understand and correctly interpreted by the target audience. This important aspect of face validation ensured that the PROM “looked right” to the interview cohort, making it probable that they will also be suitable for similar patients. The open discussions at the end of the interview encouraged patients to consider arrhythmia related problems missing from the PROM which should be included. This helped to ensure the PROM is fully inclusive.

The final tool will be used together with EQ-5D-5L (with permission from EuroQol) in a prospective study of over 450 patients at three sites to obtain more evidence of validity such as predictive and discriminant validity, and reliability. This will involve assessing measures such as reliability of the tool, and enable statistical comparison of the PROM results with EQ-5D outcomes and objective clinical measures. This will provide further evidence of the PROM tool’s psychometric performance for future research. The five year follow up will provide useful data on the long-term outcome following ablation, which will inform future work.

### Limitations

The main drawback of our approach to survey revision is the small sample involved in this process. While authors [25,26] suggest that 20 interviews are sufficient to reach data saturation, there is the risk that those involved will not be fully representative of the target population. This may be a particular issue with regard to the large number of different arrhythmias, and the corresponding differences in symptoms these can cause. Information from the prospective study will provide data from a large number of patients with various arrhythmias substrates which will ensure representation of all patient subgroups. This will not only allow further analysis to take place for validation of the tool but will also facilitate the comparison of data across the patient population.

Those patients who declined to take part in this study may have had different issues, or been more symptomatic, than those who agreed to be participants. One could also argue that the patients interviewed, who were from tertiary care medical centres which treat more complex diseases, could have been more symptomatic and not characteristic of the cardiac arrhythmia patient population in general. However, this study involved a range of patients from different geographic areas of the UK in an effort to reduce this risk.

While presenting patients with a list of predetermined symptoms and concerns may inhibit spontaneous identification of those most important to the interviewee, this was minimised as far as possible by the use of additional techniques. Face to face interviews are also highly reactive to individual personalities and rely on the skills of the researcher to elicit information from the participants. The use of a single researcher carrying out the interviews may have compounded this to introduce a natural bias; however this strategy also added a consistency to the interviews. Qualitative research of this type can be prone to error in interpreting patient responses, although probing questions aimed to minimise this, as well as our efforts to include other researchers knowledgeable in qualitative analysis who participated in the coding and analyses. Retesting the modified tool in a different subgroup of patients helped to ensure that the changes made were comprehensible and reflective of patient requirements.

## Conclusion

The interviews provided essential insight into core issues important to symptomatic arrhythmia patients with recurring themes identified; this allowed weaknesses in the tool to be addressed. Modifications were verified as appropriate via subsequent patient interviews and no new relevant themes or issues were highlighted. Feedback from the patients during this phase of the study assisted with establishing preliminary face and content validity of the PROM, and has also built on previous work to improve usability. Key issues which arose during the interviews facilitated improving clarity of wording, and the addition of items covering symptoms and the impact of the arrhythmia on patients’ daily life. Subjects completing the revised version of the tool found it to be fully comprehensive and appropriate to them as a target audience. The participants found the revised instructions to be clear and logical and the formatting was acceptable and easy to use.

There are several benefits of gaining patient views in health care, not least that patients welcome being involved and this in itself may confer health benefits [27]. There is increasing recognition that gaining patient feedback is an important aspect of measuring the efficacy and quality of healthcare, and can help support commissioning and increase accountability. Cardiac ablation aims to reduce or abolish symptoms and increase quality of life, making it an ideal area for PROM use. The use of PROMs in this patient group will provide useful data on the clinical benefit of ablation, and provide data to inform both patients and clinicians. Inclusion of the EQ-5D in this PROM will allow QALYs to be calculated, and this together with data gathered on patient reported days lost from work, hospital and GP visits will provide useful economic data.
